# A Prospective Study of Depression and Quality of Life after Kidney Transplantation

**DOI:** 10.34067/KID.0000000000000538

**Published:** 2024-08-06

**Authors:** Cecile L. Hermanns, Kate Young, Adam Parks, William M. Brooks, Rebecca J. Lepping, Robert N. Montgomery, Aditi Gupta

**Affiliations:** 1 Division of Nephrology and Hypertension, Department of Internal Medicine, University of Kansas Medical Center, Kansas City, Kansas; 2 Department of Biostatistics and Data Science, University of Kansas Medical Center, Kansas City, Kansas; 3 Department of Neurology, University of Kansas Medical Center, Kansas City, Kansas; 4 Frontiers Clinical and Translational Science Institute, University of Kansas, Kansas City, Kansas

**Keywords:** depression, kidney failure, kidney transplantation, quality of life

## Abstract

**Key Points:**

- Depression and health-related quality of life improve with kidney transplantation and is sustained beyond the immediate post-transplant period.
- The improvement in health-related quality of life, especially the physical component, differs by age and is higher in younger patients.
- Depression improves in all kidney transplantation recipients, irrespective of their age.

**Background:**

Depression and diminished health-related quality of life (HRQOL) are common in kidney failure. In this study, we investigate whether kidney transplantation (KT), the treatment of choice for kidney failure, improves depression and HRQOL across lifespan and whether this effect is sustained.

**Methods:**

In this longitudinal observational cohort study, we assessed depression and HRQOL in patients on the KT waitlist and again at 3 months and 1 year after KT. We measured depression using the Beck Depression Inventory-II (BDI-II) and HRQOL using the Kidney Disease Quality of Life Short Form Version 1.3 physical health composite score (PCS) and mental health composite score (MCS). We used linear mixed-effects models with random intercepts for patients to evaluate the effect of time, age, and KT status on BDI-II score, PCS, and MCS. For models with significant age interactions, we estimated this effect for baseline age groups.

**Results:**

We analyzed 239 longitudinal BDI-II assessments completed by 99 patients and 143 Kidney Disease Quality of Life Short Form Version 1.3 assessments completed by 59 patients (16% Black, 79% White). The BDI-II scores improved pre- to post-KT (10 pre-KT versus five post-KT, *P* < 0.001). PCS improved pre- to post-KT in younger patients, but the magnitude of change was lower with older age (*P* for interaction=0.01). In the subgroup analysis by age, there was improvement in PCS post-KT in patients younger than 60 years (*P* = 0.003 for 30–39, *P* = 0.007 for 40–49, *P* = 0.03 for 50–59). The MCS also improved from 47 pre-KT to 51 post-KT (*P* < 0.001), and the magnitude of improvement was again lower with older age (*P* for interaction=0.03).

**Conclusions:**

Depression and HRQOL improve with KT. While depression improves in all ages, the improvement in HRQOL, especially PCS, is more evident in younger patients. This improvement in depression and HRQOL is sustained until at least 1 year post-KT. These data help frame expectations for patients and transplant teams.

**Podcast:**

This article contains a podcast at https://dts.podtrac.com/redirect.mp3/www.asn-online.org/media/podcast/K360/2024_09_26_KID0000000000000538.mp3

## Visual Abstract

**Figure:**
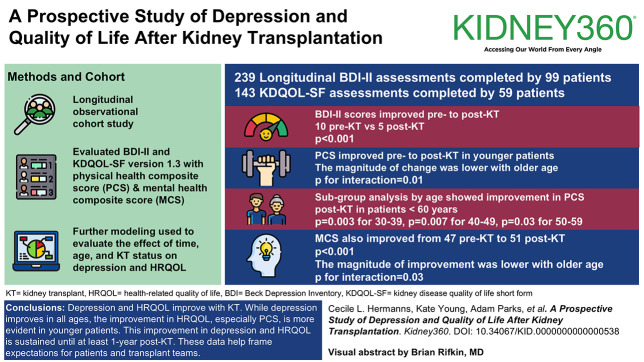

## Introduction

The prevalence of depression in patients with kidney failure is 39%, approximately double the prevalence of 18.5% in the general population.^1,2^ Kidney failure is also associated with a lower health-related quality of life (HRQOL) compared with the general population.^3^ Moreover, depression and HRQOL are often associated with each other.^4^ Both depression and low HRQOL in kidney failure are related to the negative effect of CKD on physical and social function, vocation, diet, and dialysis procedure.^5,6^ Depression and low HRQOL affect self-esteem and self-worth. Depressive symptoms in patients receiving dialysis are associated with an increase in mortality, which may be due to higher suicide rates,^7^ poorer medication adherence, withdrawal from dialysis therapy,^8–11^ and cardiovascular disease.^12^ Indeed, suicides are 84% more common in patients receiving dialysis than the general population.^7^ Dialysis withdrawal is 6.4% higher with depression, leading to a total withdrawal rate of 18% in patients with depression receiving dialysis.^7,10,11^ Overall, mortality is 50% greater in patients receiving dialysis who exhibit depressive symptoms.^6^

Kidney transplantation (KT) is the treatment of choice for patients with kidney failure. However, longitudinal data on depression and HRQOL before and after KT across lifespan are limited. In a meta-analysis by Palmer *et al.* investigating depression in CKD, there were 170 studies with 43,650 participants on dialysis, compared with only 22 studies with 4650 participants for KT recipients.^1^ Cross-sectional comparative studies indicate depression to be lower in KT recipients than in patients receiving dialysis.^9^ In the abovementioned meta-analysis, the prevalence of depression was 27% in KT recipients compared with 39% in kidney failure.^1^ However, because of the cross-sectional nature of these studies, it cannot be ascertained whether depression improves after KT or whether this difference is due to a lower prevalence of depression in patients who undergo KT. Similar to data on depression, cross-sectional studies have found that HRQOL is better in KT recipients than in patients receiving dialysis.^3,13^

Thus, the data on effect of KT on depression and HRQOL are limited, and it remains unclear whether the low prevalence of depression and better HRQOL in KT recipients is due to improvement after KT or due to careful selection of patients for KT that may limit eligibility of patients with severe depression. In addition, it is not clear whether changes in depression and HRQOL differ by age of the patient.

Transplant centers have a difficult task of estimating the benefit of a KT for a particular patient to justify the risks of undergoing a major surgery. It is thus important for transplant centers to understand how KT may affect depression and HRQOL in a potential KT recipient. In this longitudinal cohort, we assessed the changes in depression and HRQOL after KT in patients with kidney failure as an aggregate and across difference recipient ages. We also estimated the clinical relevance of these findings to assist transplant centers with decisions regarding selection and management of patients for KT. We enrolled patients waitlisted for KT and followed them prospectively. This study adds to previous knowledge with its larger sample size, analysis of age-specific changes, and longer follow-up time. We hypothesized that depression and HRQOL improve with KT, especially in younger patients where depression and HRQOL are largely due to CKD and dialysis and not as much due to other comorbidities.

## Methods

In this single-center longitudinal observational cohort study, we assessed the changes in depression (primary outcome) and HRQOL (secondary outcome) in patients with kidney failure before and after KT. We enrolled patients with kidney failure who were listed for KT. We assessed depression and HRQOL at baseline, at 3 months post-KT, and at 12 months post-KT. If a patient did not receive a KT within 1 year of the baseline assessment, we repeated the depression and HRQOL assessments to ensure that recent pre-KT data were available for comparison with post-KT scores. The study protocol was approved by the Institutional Review Board (study 13566). The clinical and research activities being reported are consistent with the principles of the Declaration of Helsinki and the Declaration of Istanbul as outlined in the “Declaration of Istanbul on Organ Trafficking and Transplant Tourism.”

### Participants

We enrolled patients waitlisted for a KT who were likely to receive a KT within a year. These included patients who were scheduled for a living donor KT had been waitlisted for KT for two or more years or were backups for recent organ offers. Patients were eligible for enrollment if they were older than 18 years and able to sign the informed consent on their own. Patients were excluded if they had hearing or visual impairment;, were unable to read, write, speak, or understand English; had uncontrolled psychosis or seizure disorder; or were currently using antipsychotics for conditions other than depression or antiepileptics. All participants signed an informed consent before initiating study procedures. Baseline demographics were obtained by self-report for all participants and included age, race, sex, ethnicity, level of education, and marital status.

### Outcome Variables

Our primary outcome was depression measured using the Beck Depression Inventory-II (BDI-II).^14^ The BDI-II is a validated screening tool consisting of 21 questions. BDI-II scores range from 0 to 63, with higher scores indicating more severe depression.^15^ BDI-II scores of 0–13 indicate no or minimal depression, 14–19 indicate mild depression, and 20–63 indicate moderate-to-severe depression.^15^

Our secondary outcomes were the physical health composite scores (PCS) and mental health composite scores (MCS) of the Kidney Disease Quality of Life Short Form Version 1.3 (KDQOL-SF).^16^ The KDQOL-SF is a 43-question long questionnaire developed and validated for kidney failure. The KDQOL-SF includes the ShortFform (SF)-12, a widely used SF measurement of HRQOL. The SF-12 assesses both physical and mental health, and consists of questions about physical functioning, role limitations due to physical health problems, pain, general health, energy and fatigue, social functioning, role limitations due to emotional problems, and mental health.^17^ The SF-12 PCS and MCS were calculated as outlined by Hays *et al*.^18^ All scores have a range of 0–100, with higher scores indicating higher quality of life.

### Statistical Analysis

We summarized baseline characteristics using means and SDs for continuous variables and frequencies for categorical variables. The BDI-II scores and KDQOL-SF subscale scores were also summarized at each time point using the means, SDs, medians, and ranges of the observed scores. We used linear mixed-effects models with random intercepts for each patient to evaluate the effect of age at baseline, race, sex, education, diabetes, time on dialysis, time since baseline, and transplant status on BDI-II score, PCS, and MCS. Model assumptions were assessed through analysis of residuals, and log transformations were applied where appropriate. The models also included an interaction between age at baseline and transplant status (pre-KT versus post-KT) and an interaction between time since baseline and transplant status. We estimated overall effects of age, transplant status, and time by averaging their effects across their corresponding interaction variables. For models with significant interactions, we estimated the effect of KT status at 1 year post-KT for age groups of 30–39, 40–49, 50–59, 60–69, and ≥70 years. We also plotted the observed BDI-II, PCS, and MCS pre-KT and post-KT by age with linear fitted pre-KT and post-KT score estimates by age, averaged across all post-baseline time points.

The correlation between post-KT eGFR and post-KT BDI-II, PCS, and MCS were also assessed. We used repeated-measures correlations to account for the within-patient associations arising from the repeated observations at 3 months post-KT and 1 year post-KT.^19^ Scatterplots for each post-KT score versus eGFR were created to visually assess these correlations. Fitted regression lines for each patient were also included in these plots to account for the repeated measurements.

## Results

Baseline characteristics are summarized in Table 1. At baseline, 99 patients completed the BDI-II: 69 had no or minimal depression, 17 had mild depression, and 13 had moderate-to-severe depression. The patients were 54.3±11.9 years old, 42% were female, 16% were Black, 78% were White, and 4% were Hispanic. Education level was high, with 35% patients reporting some college and 27% with a 4-year degree. The most common cause of kidney failure was diabetes (25%). A majority (75%) of the patients were on dialysis, and 60% of those were on hemodialysis.

**Table 1:** Baseline characteristics of the study participants by Beck Depression Inventory-II scores

Characteristic	No-Minimal Depression (*n*=69)	Mild Depression (*n*=17)	Moderate-Severe Depression (*n*=13)	Overall (*N*=99)
Age at baseline (yr), mean±SD	54.8±12.5	56.4±8.4	48.8±11.5	54.3±11.9
Follow-up time (mo), mean±SD	12.2±7.0	13.6±9.9	7.4±8.0	11.8±7.8
Female sex, *n* (%)	29 (42.0)	7 (41.2)	6 (46.2)	42 (42.4)
**Race, *n* (%)**				
Black or African American	13 (18.8)	2 (11.8)	1 (7.7)	16 (16.2)
White	53 (76.8)	13 (76.5)	12 (92.3)	78 (78.8)
Other^a^	3 (4.3)	2 (11.8)	0	5 (5.1)
**Ethnicity, *n* (%)**				
Hispanic or Latino	3 (4.3)	1 (5.9)	0	4 (4.0)
**Education, *n* (%)**				
High school diploma, no college	9 (13.0)	2 (11.8)	4 (30.8)	15 (15.2)
Some college	24 (34.8)	6 (35.3)	5 (38.5)	35 (35.4)
4-yr degree	19 (27.5)	5 (29.4)	3 (23.1)	27 (27.3)
Attended graduate school	17 (24.6)	4 (23.5)	1 (7.7)	22 (22.2)
**Marital status, *n* (%)**				
Single	13 (18.8)	2 (11.8)	3 (23.1)	18 (18.2)
Married	50 (72.5)	13 (76.5)	8 (61.5)	71 (71.7)
Divorced	5 (7.2)	1 (5.9)	1 (7.7)	7 (7.1)
Other	1 (1.4)	1 (5.9)	1 (7.7)	3 (3.0)
**Cause of kidney failure^b^, *n* (%)**				
Diabetes	19 (27.5)	4 (23.5)	2 (15.4)	25 (25.3)
Hypertension	10 (14.5)	4 (23.5)	3 (23.1)	17 (17.2)
GN	4 (5.8)	2 (11.8)	0	6 (6.1)
PKD	14 (20.3)	3 (17.6)	3 (23.1)	20 (20.2)
Other	36 (52.2)	9 (52.9)	6 (46.2)	51 (51.5)
On dialysis at baseline, *n* (%)	49 (71.0)	14 (82.4)	11 (84.6)	74 (74.7)
**Mode of dialysis, *n* (%)**				
In-home HD	3 (6.1)	0	1 (9.1)	4 (5.4)
In-center HD	29 (59.2)	8 (57.1)	3 (27.3)	40 (54.1)
PD	17 (34.7)	6 (42.9)	7 (63.6)	30 (40.5)
**Dialysis access, *n* (%)**				
Catheter	20 (40.8)	7 (50.0)	7 (63.6)	34 (45.9)
AVF	30 (61.2)	7 (50.0)	4 (36.4)	41 (55.4)
AVG	1 (2.0)	0	1 (9.1)	2 (2.7)

No-minimal depression was defined by Beck Depression Inventory-II scores 0–13, mild depression was defined by Beck Depression Inventory-II scores of 14–20, and moderate-severe depression was described as Beck Depression Inventory-II scores of 20 or more. AVF, arteriovenous fistula; AVG, arteriovenous graft; BDI-II, Beck Depression Inventory-II; PD, peritoneal dialysis; PKD, polycystic kidney disease.

a Other category represents all patients who self-identified as Native Hawaiian, Pacific Islander, Asian, other, or unknown.

b Some patients had more than one cause of kidney failure. Continuous values are presented as mean and SD. Categorical variables are presented as frequency (%).

**Figure 1:**
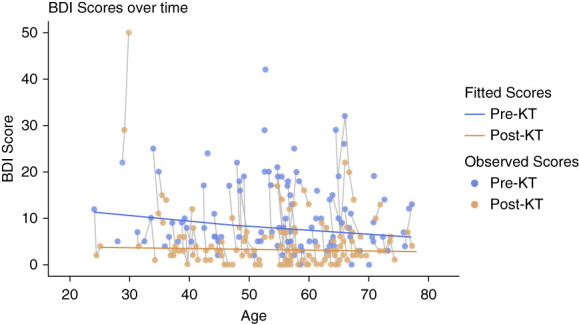
**Pretransplant and post-transplant BDI-II scores.** Scatterplots display individual participant data as a function of age and KT status. Blue dots are the observed scores for each patient pre-KT, and orange dots are the observed scores for each patient post-KT. Solid gray lines represent individual participant trajectory over time. The solid orange line represents the post-KT fitted scores by age. Distance between blue and orange lines represents the estimated group effect. The slope of the blue and orange lines represents the overall effect of age. BDI-II, Beck Depression Inventory-II; KT, kidney transplant.

Supplemental Table 1 presents the mean unadjusted BDI-II scores at baseline, 1 year after baseline (for those not transplanted within a year since baseline visit), 3 months post-KT, and 1 year post-KT, as well as the percent change in BDI-II score at 3 months and 1 year post-KT. The percent change in BDI-II at 3 months post-KT (48.3%) and at 1 year post-KT (48.6%) was greater than the minimal clinically important difference (MCID) of 17.5%.^20^ Eight patients repeated a pre-KT assessment 1 year after the baseline assessment, 69 completed the 3-month post-KT assessment, and 62 completed the 1-year post-KT assessment. Table 2 presents the linear mixed-effects model estimates for BDI-II scores. Compared with pre-KT values (baseline and 1 year after baseline), the post-KT (3 months and 1 year post-KT) BDI-II scores were significantly lower (*P* < 0.001). There was no association between the BDI-II score and age, race, sex, time since baseline, education level, history of diabetes, or time on dialysis (*P* > 0.05 for all). There was no interaction between KT status (pre-KT versus post-KT) and age or between KT status and time. Figure 1 shows the trajectory of BDI-II scores for individual patients over time and the relationship of the score with age and KT status.

**Table 2:** Linear mixed-effects model estimates assessing the association between Beck Depression Inventory-II scores and demographic and clinical variables

Effect	exp(*β*)	95% CI	*P* Value
Age (yr)	0.990	0.976 to 1.004	0.14
Race (ref: White)	0.909	0.615 to 1.343	0.63
Female (ref: Male)	0.959	0.712 to 1.292	0.78
Years of education	0.943	0.873 to 1.018	0.13
Time (yr)	1.300	0.845 to 2.001	0.23
History of diabetes (ref: no diabetes)	1.069	0.753 to 1.519	0.71
Time on dialysis (yr)	1.020	0.918 to 1.132	0.72
Post-KT (ref: pre-KT)	0.370	0.180 to 0.762	0.01
Post-KT (ref: pre-KT)×age	1.006	0.993 to 1.019	0.34
Post-KT (ref: pre-KT)×time (yr)	0.696	0.441 to 1.098	0.12
**Overall effects**			
Age at baseline (yr)^a^	0.993	0.980 to 1.005	0.25
Post-KT (ref: pre-KT)^b^	0.424	0.317 to 0.568	<0.001
Time since baseline (yr)^a^	1.084	0.844 to 1.394	0.53

Effects are modeled for log(BDI-II score+1). BDI-II, Beck Depression Inventory-II; CI, confidence interval; KT, kidney transplantation.

a Effect averaged across pre- and post-KT status.

b Effect averaged across 3-month and 1-year post-KT status.

**Figure 2:**
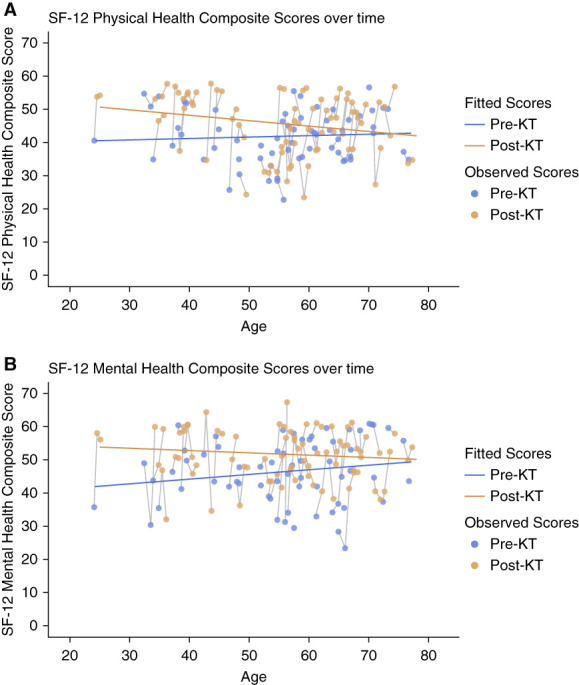
**Pre-transplant and post-transplant Short Form-12 scores.** (A) Physical health composite score (PCS) and (B) mental health composite score (MCS). Scatterplots display individual participant data as a function of age and KT status. Blue dots are the observed scores for each patient pre-KT, and orange dots are the observed scores for each patient post-KT. Solid gray lines represent individual participant trajectory over time. The solid orange line represents the post-KT fitted scores by age. Distance between blue and orange lines represents the estimated group effect. The slope of the blue and orange lines represents the overall effect of age. MCS, mental health composite score; PCS, physical health composite score; SF, Short Form.

Supplemental Table 2, A and B, presents the demographic characteristics by baseline PCS and MCS of the 59 patients who completed the baseline KDQOL-SF. Fifty-nine patients completed the PCS and MCS assessment at baseline, five repeated a pre-KT assessment 1 year after the baseline assessment, 43 completed the 3-month post-KT assessment, and 35 completed the 1-year post-KT assessment. Table 3 presents the linear mixed-effects model estimates for PCS. Overall, average PCS did not change pre- to post-KT. There was an interaction between KT status and age (*P* value for interaction=0.01) and between KT status and time (*P* value for interaction=0.04). However, there was no effect of age or time on PCS averaged across pre- and post-KT status. The effects of both age and time on the PCS differed between pre-KT versus post-KT scores. There was also a significant effect of history of diabetes on PCS (*P* = 0.04). There was no association between education level, time on dialysis, sex, or race and PCS (*P* > 0.05 for all). The observed pre-KT PCS and post-KT PCS, plotted by age of the patient, are shown in Figure 2A. The estimated change in mean PCS for patients younger than 60 years was also higher than the MCID of 5.7.^21^

**Table 3:** Linear mixed-effects model estimates assessing the association between Short Form-12 physical health composite score and demographic and clinical variables

Effect	*β*	95% CI	*P* Value
Age (yr)	0.042	−0.140 to 0.223	0.65
Race (ref: White)	1.609	−3.506 to 6.724	0.54
Female (ref: Male)	1.723	−2.260 to 5.705	0.40
Years of education	0.227	−0.827 to 1.282	0.67
Time (yr)	−1.751	−7.210 to 3.707	0.53
History of diabetes (ref: no diabetes)	−4.820	−9.430 to −0.209	0.04
Time on dialysis (yr)	−0.165	−1.715 to 1.384	0.83
Post-KT (ref: pre-KT)	10.709	1.484 to 19.935	0.02
Post-KT (ref: pre-KT)×age	−0.203	−0.363 to −0.044	0.01
Post-KT (ref: pre-KT)×time (yr)	5.938	0.187 to 11.688	0.04
**Overall effects**			
Age at baseline (yr)^a^	−0.060	−0.223 to 0.104	0.47
Post-KT (ref: pre-KT)^b^	3.037	−0.883 to 6.957	0.13
Time since baseline (yr)^a^	1.218	−2.082 to 4.517	0.47

CI, confidence interval; KT, kidney transplantation; PCS, physical health composite score.

a Effect averaged across pre- and post-KT status.

b Effect averaged across 3-month and 1-year post-KT status.

MCS improved post-KT (*P* < 0.001) (Table 4). MCS was also associated with time since baseline assessment (*P* = 0.02). As with the PCS, there was a significant interaction between age and KT status (*P* value for interaction=0.03). There was also a significant interaction between time since baseline and KT status (*P* value for interaction=0.03). Figure 2B shows the observed pre- and post-KT MCS, plotted by age of the patient. MCS was not associated with history of diabetes, years of education, sex, race, or time on dialysis (*P* > 0.05 for all). The change in mean MCS was higher than the MCID of 9.2.^21^

**Table 4:** Linear mixed-effects model estimates assessing the association between Short Form-12 mental health composite scores and demographic and clinical variables

Effect	*β*	95% CI	*P* Value
Age (yr)	0.141	−0.043 to 0.324	0.13
Race (ref: White)	0.728	−4.137 to 5.592	0.77
Female (ref: Male)	0.883	−2.862 to 4.628	0.64
Years of education	0.039	−0.957 to 1.035	0.94
Time (yr)	−7.718	−14.121 to −1.314	0.02
History of diabetes (ref: no diabetes)	2.165	−2.182 to 6.512	0.33
Time on dialysis (yr)	−0.179	−1.651 to 1.292	0.81
Post-KT (ref: pre-KT)	16.374	5.270 to 27.479	0.004
Post-KT (ref: pre-KT)×age	−0.209	−0.402 to −0.017	0.03
Post-KT (ref: pre-KT)×time (yr)	7.518	0.673 to 14.362	0.03
**Overall effects**			
Age at baseline (yr)^a^	0.036	−0.117 to 0.189	0.64
Post-KT (ref: pre-KT)^b^	9.302	4.735 to 13.868	<0.001
Time since baseline (yr)^a^	−3.959	−7.793 to −0.124	0.04

CI, confidence interval; KT, kidney transplantation; MCS, mental health composite score.

a Effect averaged across pre- and post-KT status.

b Effect averaged across 3-month and 1-year post-KT status.

**Figure 3:**
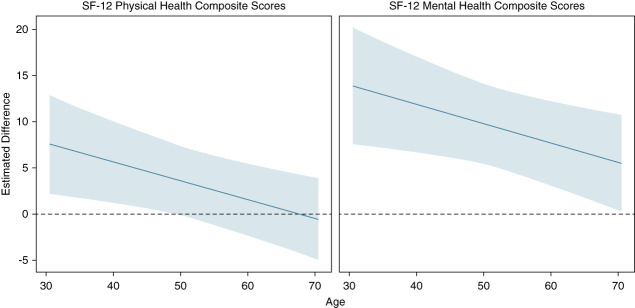
**Estimated differences in post- and pre-kidney transplantation SF-12 PCS and MCS.** Solid line represents the estimated differences as a function of age. Pale margins represent the confidence interval. The dotted line represents an estimated difference of 0.

Owing to significant interaction effects of age in the models for PCS and MCS, least square mean differences between post-KT and pre-KT scores were calculated for varying ages ranging from 30 to 70 years. Because the mean follow-up time was close to 1 year, the post-KT scores were estimated at 1 year since baseline. These estimates and 95% confidence intervals are presented in Table 5. Estimated differences and 95% confidence bands are plotted by age at 1 year after baseline in Figure 3. PCS improved post-KT for ages 30–39, 40–49, and 50–59 years (*P* = 0.003 for age 30–39 years, *P* = 0.007 for age 40–49 years, *P* = 0.03 for age 50–59 years). However, this improvement was lower for patients 60 years and older and was not statistically significant. The improvement in MCS remained statistically significant across all age groups. However, the degree of improvement decreased with increase in age.

**Table 5:** Estimated post-KT versus pre-KT differences in Short Form-12 physical health composite score and mental health composite score composite scores at 1 year after baseline

Age at Baseline	Physical Health Score			Mental Health Score		
	Estimate	95% CI	*P* Value	Estimate	95% CI	*P* Value
30–39	10.548	3.640 to 17.456	0.003	17.611	9.445 to 25.777	<0.001
40–49	8.515	2.404 to 14.625	0.007	15.517	8.330 to 22.704	<0.001
50–59	6.482	0.805 to 12.158	0.03	13.423	6.771 to 20.076	<0.001
60–69	4.448	−1.242 to 10.139	0.12	11.329	4.660 to 17.999	0.001
≥70	2.415	−3.733 to 8.564	0.44	9.236	2.002 to 16.470	0.01

CI, confidence interval; KT, kidney transplantation.

Supplemental Figure 1 and Supplemental Table 3 present the correlation between the post-KT eGFR and post-KT BDI-II, PCS, and MCS. There was no significant correlation between post-KT eGFR and post-KT BDI-II, PCS, or MCS (*P* > 0.05 for all).

## Discussion

We found that both depression and HRQOL improved post-KT across all ages, and this improvement was sustained for at least 1 year post-KT. However, there was an age-associated difference in change in HRQOL after KT; the improvement in HRQOL diminished with older age.

Previous cross-sectional studies have assessed depression in patients awaiting KT and in KT recipients.^1^ Our study also found the prevalence of depression to be lower post-KT; 30% of participants had at least mild depression pre-KT compared with 7% post-KT. Robiner *et al.* found a similar pre-KT prevalence of 33%.^8^ Specifically, they found that 12% had mild depression and 10% had moderate depression pre-KT. In our study, 17% had mild depression and 13% had moderate-severe depression pre-KT. By contrast, Wu *et al.* found a prevalence of 91% using the Self-Rating Depression Scale.^22^ The difference in assessment tools may contribute to the different prevalence rates in published studies. The high level of education in our cohort may also explain the lower prevalence when compared with the study by Wu *et al.* Indeed, lower education is associated with higher depression in kidney failure,^23^ and our cohort had a high level of education.

BDI-II scores improved post-KT. The improvement in depression seen in our study is consistent with and validates the results of some prior studies. In their prospective study of 28 patients who received a KT, Robiner *et al.* did not find a statistical improvement in BDI-II scores, whereas the Millon Clinical Multiaxial Inventory III—Major Depression Scale scores improved from pre-KT to 6 months post-KT.^8^ The improvement in BDI-II scores (9±6 pre-KT to 8±7 post-KT) was similar to the improvement observed in our study (10±8 pre-KT to 5±7 post-KT), but the smaller sample size may have limited the statistical significance in change in BDI-II. Another prospective study of 128 patients by Wu *et al.* showed a significant decrease in depression at 3 months post-KT.^22^ Before transplantation, 90.6% of recipients were depressed, which decreased to 44.5% at 90 days post-KT.^22^

We did not find any association between depression and age or diabetes. Previous studies have observed an association between depression and history of diabetes in KT recipients. In a retrospective study of over 47,000 KT recipients, Dobbels *et al.* found that patients with diabetes as the primary cause of kidney failure were 51% more likely to be depressed post-KT.^24^ One potential explanation is that diabetes-related health issues can increase depression. Dobbels *et al.* also found that KT recipients older than 65 years were 14% less likely to be depressed post-KT than those aged 18–24 years, possibly because of improved coping in older patients.^24^ However, we did not observe any association between age and depression or an interaction between age and KT status on change in depression pre-KT to post-KT.

We also assessed HRQOL and observed that HRQOL improved after KT. The mean PCS and MCS increased from 42±8 to 49±7 and 47±9 to 51±8, respectively, pre- to post-KT. Our results support the findings of cross-sectional studies that found HRQOL to be lower in patients receiving dialysis than in KT recipients.^13,25^ Our results are also consistent with the finding of other studies that HRQOL is generally lower in patients receiving dialysis than the general population, with both baseline PCS and MCS lower than the mean score of the PCS and MCS, as determined by the SF-36, in the general population.^26^ Although we did not see an association between depression and diabetes, we did observe an association between PCS and diabetes, suggesting that diabetes-related complications may negatively affect physical health in kidney failure.

We assessed the association between age and improvement in HRQOL. In our study, improvement in PCS and MCS decreased with older age. Another study showed a higher level of physical functioning after KT in patients younger than 30 years.^27^ In a prospective study of 93 patients who underwent a KT, improvements in both physical and mental aspects of HRQOL after KT were lower for older patients, specifically among those aged 55 years and older.^28^ Another cross-sectional study found a lower physical HRQOL with increasing age in KT recipients.^29^

Importantly, in our study, the percent change in BDI-II and MCS was greater than the MCID determined by prior studies.^20,21^ The estimated change in mean PCS for patients younger than 60 years was also higher than the MCID.^21^ Other strengths of this study include the longitudinal follow-up for 1 year post-KT, a large sample size compared with previous studies, adjustment for clinically relevant covariates, such as history of diabetes; level of education; race; sex; time on dialysis; inclusion of interactions between age, time, and transplant status; and clinical interpretation using the MCID. Our study also provides measures for both depression and HRQOL at multiple time points before and after KT. However, a single center, a high level of education, and selection of patients listed for KT and likely to undergo KT within a year may affect generalizability to all patients with kidney failure. This study does not provide information about all patients with kidney failure. Patients with kidney failure who have severe depression and poor HRQOL are often not referred or listed for KT and were thus not included in this analysis.

In conclusion, depression is common among patients with kidney failure listed for KT and depression improves post-KT, regardless of age, and the improvement in depression is both statistically and clinically significant. Many patients waitlisted for KT report experiencing lower than average HRQOL. PCS improves after KT in patients younger than 60 years, but not in those older than 60 years. MCS improves after KT in all age groups. While both depression and HRQOL can improve with KT, the improvement in physical HRQOL may be limited for older patients. These data help frame expectations for patients and transplant teams.

## Supplementary Material

SUPPLEMENTARY MATERIAL

## Data Availability

All data are included in the manuscript and/or supporting information.

## Supplemental Material

Supplemental Table 1. Beck Depression Inventory-II (BDI)-II, SF-12 physical health composite score (PCS), and SF-12 mental health composite score (MCS) pre- and post-kidney transplant (KT).

Supplemental Table 2. Demographic characteristics by baseline SF-12 (A) physical health composite score (PCS) and (B) mental health composite score (MCS).

Supplemental Table 3. Estimated repeated-measures correlation between post-kidney transplant (KT) eGFR and Beck Depression Inventory-II (BDI-II), SF-12 physical health composite score (PCS), and SF-12 mental health composite score (MCS).

Supplemental Figure 1. Post-kidney transplant (KT) (A) Beck Depression Inventory-II (BDI-II), (B) SF-12 physical health composite score (PCS), and (C) SF-12 mental health composite score (MCS) versus eGFR.
