# Supplementary material for: A Prospective Study of Depression and Quality of Life after Kidney Transplantation
Source: Kidney360. 2024 Aug 6;5(9):1350–8. doi: 10.34067/KID.0000000000000538 (PMC11441810; doi:10.34067/KID.0000000000000538)
Supplement: SUPPLEMENTARY MATERIAL [file kidney360-5-1350-s001.pdf]

ASN American Society of Nephrology
JASN Journal of the American Society of Nephrology
KIDNEY360 Accessing Our World From Every Angle
CJASN Clinical Journal of the American Society of Nephrology

ASN Journal Disclosure Form

As per ASN journal policy, I have disclosed any financial relationships or commitments I have held in the past 36 months as included below. I have listed my Current Employer below to indicate there is a relationship requiring disclosure. If no relationship exists, my Current Employer is not listed.

W. Brooks reports the following:
Employer: University of Kansas Medical Center

I understand that the information above will be published within the journal article, if accepted, and that failure to comply and/or to accurately and completely report the potential financial conflicts of interest could lead to the following: 1) Prior to publication, article rejection, or 2) Post-publication, sanctions ranging from, but not limited to, issuing a correction, reporting the inaccurate information to the authors' institution, banning authors from submitting work to ASN journals for varying lengths of time, and/or retraction of the published work.

Name: William M. Brooks
Manuscript ID: K360-2024-000173R1
Manuscript Title: A Prospective Study of Depression and Quality of Life After Kidney Transplantation
Date of Completion: July 31, 2024
Disclosure Updated Date: March 19, 2024

ASN American Society of Nephrology | JASN Journal of the American Society of Nephrology | KIDNEY360 Accessing Our World From Every Angle | CJASN Clinical Journal of the American Society of Nephrology

ASN Journal Disclosure Form

As per ASN journal policy, I have disclosed any financial relationships or commitments I have held in the past 36 months as included below. I have listed my Current Employer below to indicate there is a relationship requiring disclosure. If no relationship exists, my Current Employer is not listed.

A. Gupta reports the following:
Employer: University of Kansas Medical Center; Consultancy: Novartis Pharmaceuticals; Research Funding: NIH, Novartis pharmaceuticals, Veloxis pharmaceuticals; Honoraria: Uptodate; Advisory or Leadership Role: National kidney foundation- member of regional medical advisory board; Kidney Medicine- editorial board. No payment was received through any of these positions.; and Speakers Bureau: Bayer Pharmaceuticals.

I understand that the information above will be published within the journal article, if accepted, and that failure to comply and/or to accurately and completely report the potential financial conflicts of interest could lead to the following: 1) Prior to publication, article rejection, or 2) Post-publication, sanctions ranging from, but not limited to, issuing a correction, reporting the inaccurate information to the authors' institution, banning authors from submitting work to ASN journals for varying lengths of time, and/or retraction of the published work.

Name: Aditi Gupta
Manuscript ID: K360-2024-000173R1
Manuscript Title: A Prospective Study of Depression and Quality of Life After Kidney Transplantation
Date of Completion: July 9, 2024
Disclosure Updated Date: July 9, 2024

ASN Journal Disclosure Form

As per ASN journal policy, I have disclosed any financial relationships or commitments I have held in the past 36 months as included below. I have listed my Current Employer below to indicate there is a relationship requiring disclosure. If no relationship exists, my Current Employer is not listed.

C. Hermanns has nothing to disclose.

I understand that the information above will be published within the journal article, if accepted, and that failure to comply and/or to accurately and completely report the potential financial conflicts of interest could lead to the following: 1) Prior to publication, article rejection, or 2) Post-publication, sanctions ranging from, but not limited to, issuing a correction, reporting the inaccurate information to the authors' institution, banning authors from submitting work to ASN journals for varying lengths of time, and/or retraction of the published work.

Name: Cecile Hermanns
Manuscript ID: K360-2024-000173R1
Manuscript Title: A Prospective Study of Depression and Quality of Life After Kidney Transplantation
Date of Completion: July 9, 2024
Disclosure Updated Date: July 9, 2024

ASN American Society of Nephrology | JASN Journal of the American Society of Nephrology | KIDNEY360 Accessing Our World From Every Angle | CJASN Clinical Journal of the American Society of Nephrology

ASN Journal Disclosure Form

As per ASN journal policy, I have disclosed any financial relationships or commitments I have held in the past 36 months as included below. I have listed my Current Employer below to indicate there is a relationship requiring disclosure. If no relationship exists, my Current Employer is not listed.

R. Lepping reports the following:
Employer: University of Kansas Medical Center; Research Funding: KUMC; National Institutes of Health; Novartis; Veloxis;; and Advisory or Leadership Role: National Alzheimer's Coordinating Center Grants Reviewer, honorarium.

I understand that the information above will be published within the journal article, if accepted, and that failure to comply and/or to accurately and completely report the potential financial conflicts of interest could lead to the following: 1) Prior to publication, article rejection, or 2) Post-publication, sanctions ranging from, but not limited to, issuing a correction, reporting the inaccurate information to the authors' institution, banning authors from submitting work to ASN journals for varying lengths of time, and/or retraction of the published work.

Name: Rebecca J. Lepping
Manuscript ID: K360-2024-000173R1
Manuscript Title: A Prospective Study of Depression and Quality of Life After Kidney Transplantation
Date of Completion: July 8, 2024
Disclosure Updated Date: March 4, 2024

ASN American Society of Nephrology
JASN Journal of the American Society of Nephrology
KIDNEY360 Accessing Our World From Every Angle
CJASN Clinical Journal of the American Society of Nephrology

ASN Journal Disclosure Form

As per ASN journal policy, I have disclosed any financial relationships or commitments I have held in the past 36 months as included below. I have listed my Current Employer below to indicate there is a relationship requiring disclosure. If no relationship exists, my Current Employer is not listed.

N. Montgomery reports the following:
Employer: University of Kansas Medical Center

I understand that the information above will be published within the journal article, if accepted, and that failure to comply and/or to accurately and completely report the potential financial conflicts of interest could lead to the following: 1) Prior to publication, article rejection, or 2) Post-publication, sanctions ranging from, but not limited to, issuing a correction, reporting the inaccurate information to the authors' institution, banning authors from submitting work to ASN journals for varying lengths of time, and/or retraction of the published work.

Name: Neal Montgomery
Manuscript ID: K360-2024-000173R1
Manuscript Title: A Prospective Study of Depression and Quality of Life After Kidney Transplantation
Date of Completion: July 19, 2024
Disclosure Updated Date: June 11, 2024

ASN American Society of Nephrology | JASN Journal of the American Society of Nephrology | KIDNEY360 Accessing Our World From Every Angle | CJASN Clinical Journal of the American Society of Nephrology

ASN Journal Disclosure Form

As per ASN journal policy, I have disclosed any financial relationships or commitments I have held in the past 36 months as included below. I have listed my Current Employer below to indicate there is a relationship requiring disclosure. If no relationship exists, my Current Employer is not listed.

A. Parks reports the following:
Employer: University of Kansas Medical Center

I understand that the information above will be published within the journal article, if accepted, and that failure to comply and/or to accurately and completely report the potential financial conflicts of interest could lead to the following: 1) Prior to publication, article rejection, or 2) Post-publication, sanctions ranging from, but not limited to, issuing a correction, reporting the inaccurate information to the authors' institution, banning authors from submitting work to ASN journals for varying lengths of time, and/or retraction of the published work.

Name: Adam Parks
Manuscript ID: K360-2024-000173R1
Manuscript Title: A Prospective Study of Depression and Quality of Life After Kidney Transplantation
Date of Completion: July 10, 2024
Disclosure Updated Date: July 10, 2024

ASN American Society of Nephrology | JASN Journal of the American Society of Nephrology | KIDNEY360 Accessing Our World From Every Angle | CJASN Clinical Journal of the American Society of Nephrology

ASN Journal Disclosure Form

As per ASN journal policy, I have disclosed any financial relationships or commitments I have held in the past 36 months as included below. I have listed my Current Employer below to indicate there is a relationship requiring disclosure. If no relationship exists, my Current Employer is not listed.

K. Young reports the following:
Employer: University of Kansas Medical Center; and Ownership Interest: ICON plc.

I understand that the information above will be published within the journal article, if accepted, and that failure to comply and/or to accurately and completely report the potential financial conflicts of interest could lead to the following: 1) Prior to publication, article rejection, or 2) Post-publication, sanctions ranging from, but not limited to, issuing a correction, reporting the inaccurate information to the authors' institution, banning authors from submitting work to ASN journals for varying lengths of time, and/or retraction of the published work.

Name: Kate J. Young
Manuscript ID: JASN-2024-000520R1
Manuscript Title: Changes in Olfactory and Cognitive Function after Kidney Transplantation
Date of Completion: May 28, 2024
Disclosure Updated Date: March 25, 2024
