# Supplementary material for: A Prospective Study of Depression and Quality of Life after Kidney Transplantation
Source: Kidney360. 2024 Aug 6;5(9):1350–8. doi: 10.34067/KID.0000000000000538 (PMC11441810; doi:10.34067/KID.0000000000000538)
Supplement: SUPPLEMENTARY MATERIAL [file kidney360-5-1350-s002.pdf]

Supplemental Material

Supplemental Table 1. Beck Depression Inventory-II (BDI)-II, SF-12 physical health composite score (PCS), and SF-12 mental health composite score (MCS) scores pre- and post-kidney transplant (KT).

Supplemental Table 2. Demographic characteristics by baseline SF-12 a) physical health composite score (PCS) and b) mental health composite score (MCS).

Supplemental Table 3. Estimated Repeated Measures Correlation between post-kidney transplant (KT) estimated glomerular filtration rate (eGFR) and Beck Depression Inventory-II (BDI-II), SF-12 Physical Health Composite Score (PCS), and SF-12 Mental Health Composite Score (MCS).

Supplemental Figure 1: Post-kidney transplant (KT) a) Beck Depression Inventory-II (BDI-II), b) SF-12 Physical Health Composite Score (PCS), and c) SF-12 Mental Health Composite Score (MCS) scores versus estimated glomerular filtration rate (eGFR).

Supplemental Table 1: Beck Depression Inventory-II (BDI)-II, SF-12 physical health composite score (PCS), and SF-12 mental health composite score (MCS) scores pre- and post-kidney transplant (KT).

	<1-year pre-KT	Baseline	3-months post-KT	% change (baseline to 3-months post-KT)	1-year post-KT	% change from (baseline to 1 year post-KT)
BDI-II	14.2 ± 8.8	10.4 ± 7.8	5.1 ± 5.2	-48.3%	5.0 ± 7.4	-48.6%
PCS	39.3 ± 11.2	41.8 ± 7.5	43.1 ± 9.7	+3.8%	48.8 ± 7.4	+16.9%
MCS	37.3 ± 11.3	47.1 ± 9.2	51.7 ± 7.6	+10.8%	51.3 ± 7.5	+8.1%

All values are presented as mean ± SD.
KT; kidney transplant. BDI-II; Beck Depression Inventory. PCS; Physical Health Composite Score. MCS; Mental Health Composite Score.
For BDI-II scores, at baseline n=99, at 1 year pre-KT n=8, at 3 months post-KT n=69, and at 1-year post-KT n=62.
For PCS and MCS scores, at baseline n=59, at 1 year pre-KT n=5, at 3 months post-KT n=43, and at 1-year post-KT n=35.

Supplemental Table 2a: Demographic characteristics by baseline SF-12 physical health composite score (PCS).

Characteristic	Baseline PCS below median[1] (N=29)	Baseline PCS above median[1] (N=30)	All (N=59)
Age at baseline, mean ± SD	53.7±11.5	56.8±12.4	55.2±11.9
Follow-up time (months), mean ± SD	13.1±8.6	11.5±6.9	12.3±7.7
Female sex, n (%)	11 (37.9)	15 (50.0)	26 (44.1)
Race, n (%)			
Black or African American	4 (13.8)	5 (16.7)	9 (15.3)
White	24 (82.8)	23 (76.7)	47 (79.7)
Other [2]	1 (3.4)	2 (6.7)	3 (5.1)
Ethnicity, n (%)			
Hispanic or Latino	0	3 (10.0)	3 (5.1)
Education, n (%)			
High school diploma, no college	4 (13.8)	3 (10.0)	7 (11.9)
Some college	9 (31.0)	13 (43.3)	22 (37.3)
4-yr degree	8 (27.6)	8 (26.7)	16 (27.1)
Attended graduate school	8 (27.6)	6 (20.0)	14 (23.7)
Marital Status, n (%)			
Single	6 (20.7)	4 (13.3)	10 (16.9)
Married	18 (62.1)	24 (80.0)	42 (71.2)
Divorced	4 (13.8)	2 (6.7)	6 (10.2)
Other	1 (3.4)	0	1 (1.7)
Cause of kidney failure[3], n (%)			
Diabetes	8 (27.6)	11 (36.7)	19 (32.2)
Hypertension	3 (10.3)	5 (16.7)	8 (13.6)
Glomerulonephritis	4 (13.8)	1 (3.3)	5 (8.5)
PKD	4 (13.8)	7 (23.3)	11 (18.6)
Other	17 (58.6)	12 (40.0)	29 (49.2)
On dialysis at baseline, n (%)	22 (75.9)	19 (63.3)	41 (69.5)
Mode of dialysis, n (%)			
In-center HD	12 (54.5)	13 (68.4)	25 (61.0)
PD	10 (45.5)	6 (31.6)	16 (39.0)
Dialysis access, n (%)			
Catheter	10 (45.5)	9 (47.4)	19 (46.3)
AVF	12 (54.5)	10 (52.6)	22 (53.7)
AVG	0	1 (5.3)	1 (2.4)

[1]Median PCS = 42.3.
[2]Other category represents all patients who self-identified as Native Hawaiian, Pacific Islander, Asian, other, or unknown.
[3]Some patients had more than one cause of kidney failure.
Continuous values are presented as mean and standard deviation (SD). Categorical variables are presented as frequency (%).
PCS; SF-12 Physical Health Composite Score, PKD; polycystic kidney disease, HD; hemodialysis, PD; peritoneal dialysis, AVF; arteriovenous fistula, AVG; arteriovenous graft, eGFR; estimated glomerular filtration rate

Supplemental Table 2b: Demographic characteristics by baseline SF-12 mental health composite score (MCS).

Characteristic	Baseline MCS below median[1] (N=29)	Baseline MCS above median[1] (N=30)	All (N=59)
Age (years), mean ± SD	53.9±12.4	56.6±11.5	55.2±11.9
Follow-up time (months), mean ± SD	12.0±8.6	12.5±7.0	12.3±7.7
Female sex, n (%)	12 (41.4)	14 (46.7)	26 (44.1)
Race, n (%)			
Black or African American	2 (6.9)	7 (23.3)	9 (15.3)
White	26 (89.7)	21 (70.0)	47 (79.7)
Other [2]	1 (3.4)	2 (6.7)	3 (5.1)
Ethnicity, n (%)			
Hispanic or Latino	0	3 (10.0)	3 (5.1)
Education, n (%)			
High school diploma, no college	1 (3.4)	6 (20.0)	7 (11.9)
Some college	11 (37.9)	11 (36.7)	22 (11.9)
4-yr degree	11 (37.9)	5 (16.7)	16 (27.1)
Attended graduate school	6 (20.7)	8 (26.7)	14 (23.7)
Marital status, n (%)			
Single	5 (17.2)	5 (16.7)	10 (16.9)
Married	20 (69.0)	22 (73.3)	42 (71.2)
Divorced	3 (10.3)	3 (10.0)	6 (10.2)
Other	1 (3.4)	0	1 (1.7)
Cause of kidney failure[2], n (%)			
Diabetes	8 (27.6)	11 (36.7)	19 (32.2)
Hypertension	3 (10.3)	5 (16.7)	8 (13.6)
Glomerulonephritis	3 (10.3)	2 (6.7)	5 (8.5)
PKD	6 (20.7)	5 (16.7)	11 (18.6)
Other	14 (48.3)	15 (50.0)	29 (49.2)
On dialysis at baseline, n (%)	19 (65.5)	22 (73.3)	41 (69.5)
Mode of dialysis, n (%)			
In-center HD	10 (52.6)	15 (68.2)	25 (61.0)
PD	9 (47.4)	7 (31.8)	16 (39.0)
Dialysis access, n (%)			
Catheter	11 (57.9)	8 (36.4)	19 (46.3)
AVF	9 (47.4)	13 (59.1)	22 (53.7)
AVG	0	1 (4.5)	1 (2.4)

[1]Median MCS = 47.8.
[2]Other category represents all patients who self-identified as Native Hawaiian, Pacific Islander, Asian, other, or unknown.
[3]Some patients had more than one cause of kidney failure.
Continuous values are presented as mean and standard deviation (SD). Categorical variables are presented as frequency (%).
MCS; SF-12 Mental Health Composite Score, PKD; polycystic kidney disease, HD; hemodialysis, PD; peritoneal dialysis, AVF; arteriovenous fistula, AVG; arteriovenous graft, eGFR; estimated glomerular filtration rate.

Supplemental Table 3: Estimated Repeated Measures Correlation between post-kidney transplant (KT) estimated glomerular filtration rate (eGFR) and Beck Depression Inventory-II (BDI-II), SF-12 Physical Health Composite Score (PCS), and SF-12 Mental Health Composite Score (MCS).

	Correlation	95% CI	p-value
BDI-II	-0.023	-0.275, 0.233	0.86
PCS	-0.050	-0.382, 0.293	0.78
MCS	0.132	-0.216, 0.450	0.46

BDI-II; Beck Depression Inventory. PCS; Physical Health Composite Score. MCS; Mental Health Composite Score.
For BDI-II scores, n=73.
For PCS and MCS scores, n=46.

Supplemental Figure 1: Post-kidney transplant (KT) a) Beck Depression Inventory-II (BDI-II), b) SF-12 Physical Health Composite Score (PCS), and c) SF-12 Mental Health Composite Score (MCS) scores versus estimated glomerular filtration rate (eGFR).

a.

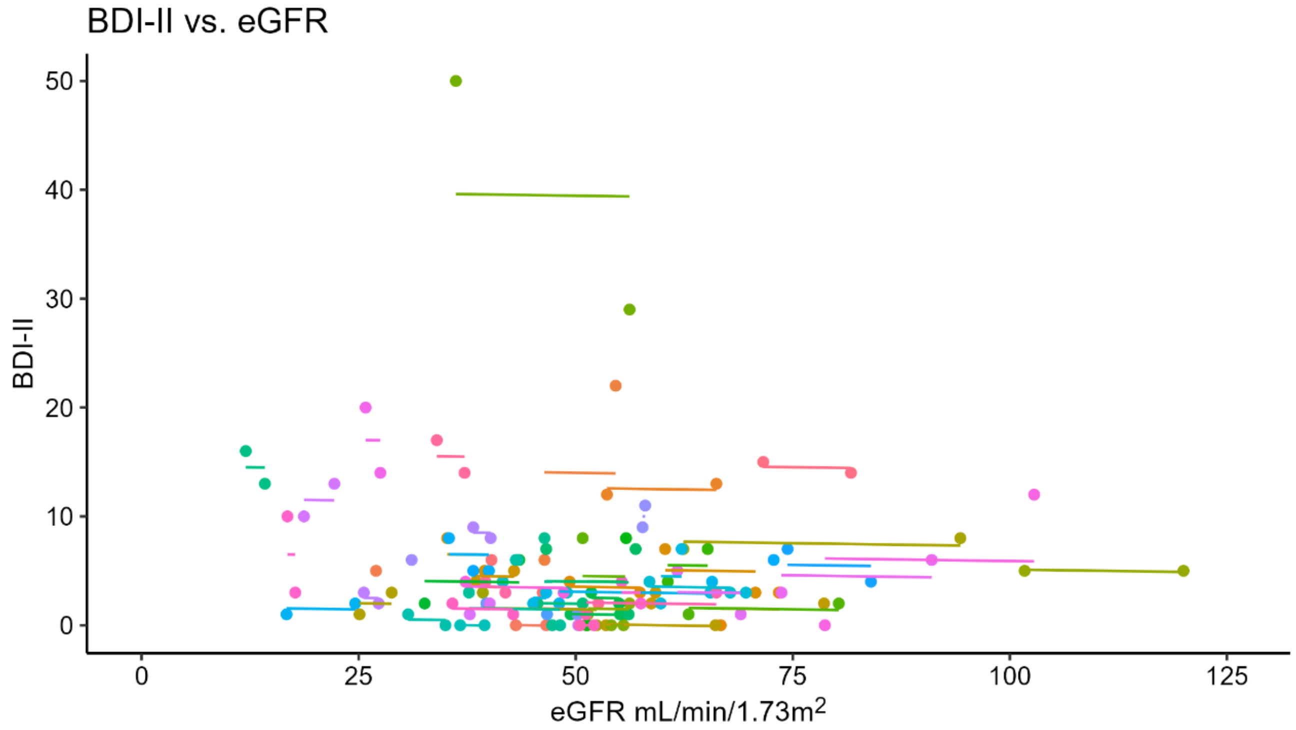

b.

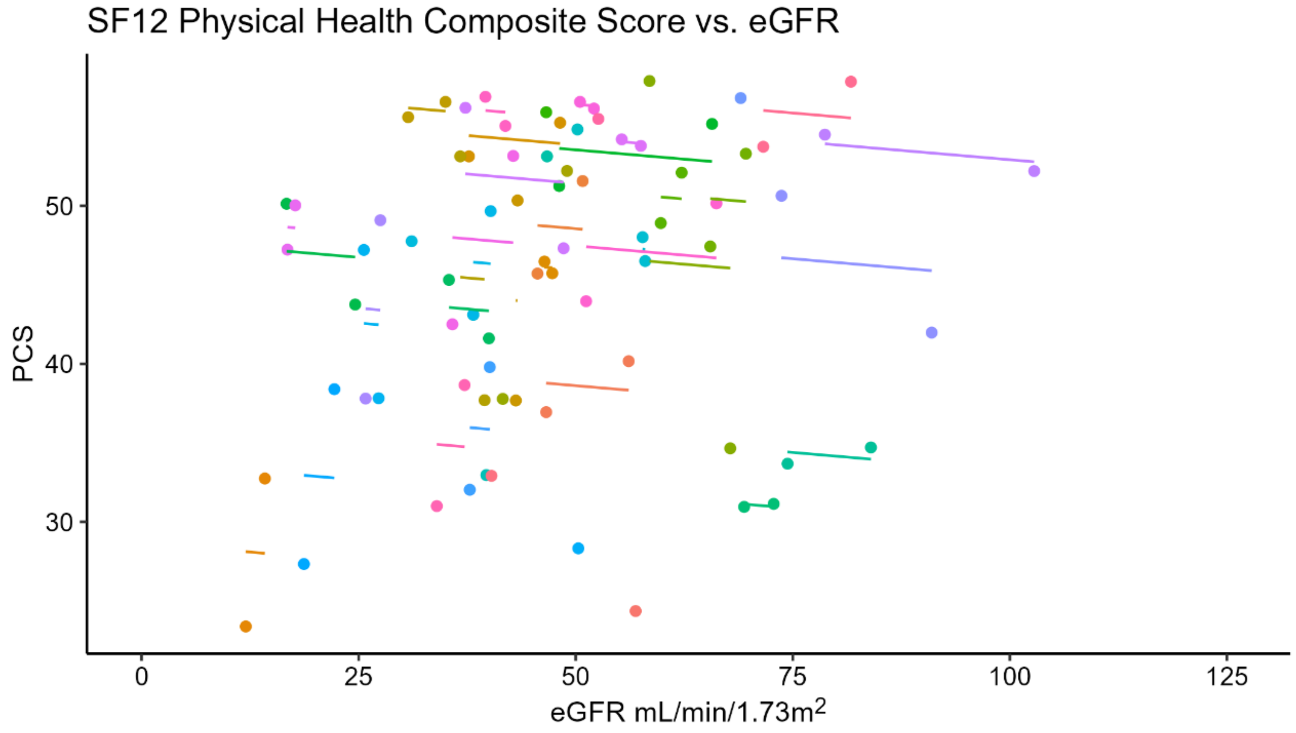

c.

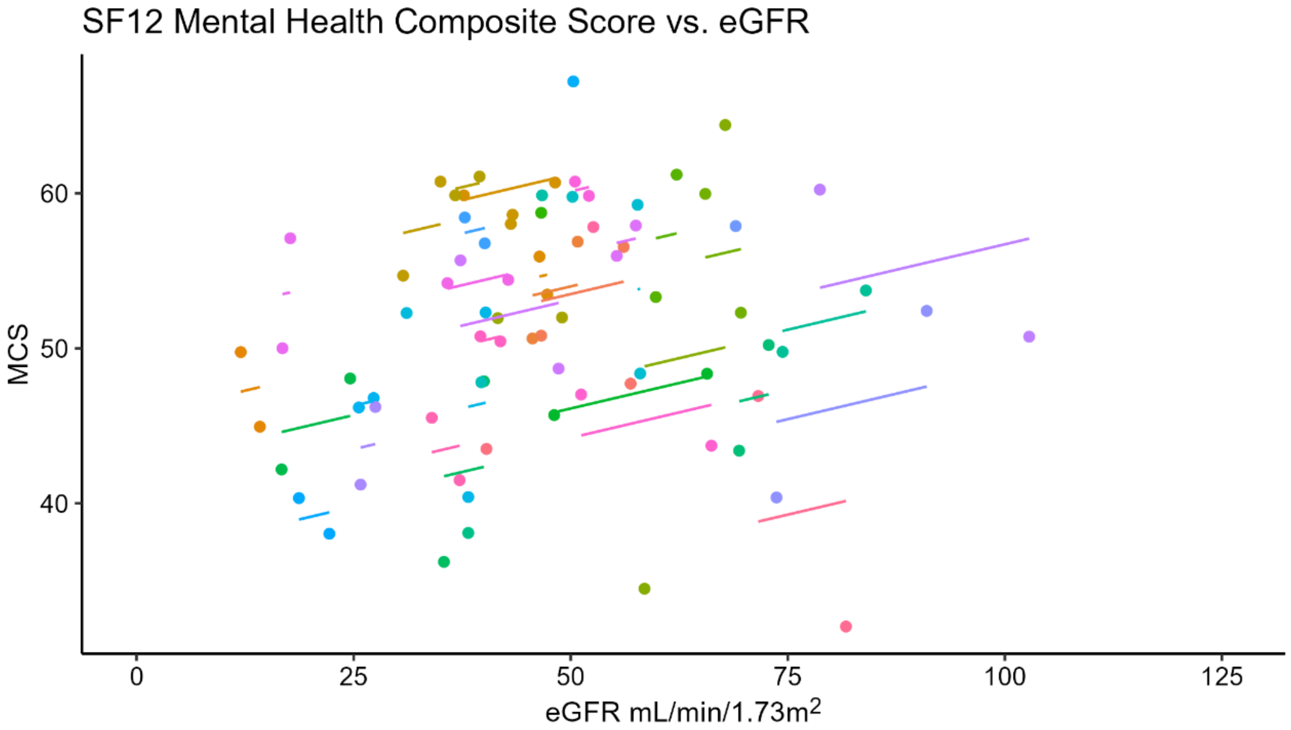

Scatterplots display individual participant data as a function of eGFR. Dots are the observed post-KT scores for each patient. Lines represent fitted regression lines for patients who had repeat measurements post-KT.
BDI-II; Beck Depression Inventory-II. MCS; SF-12 Mental Health Composite Score. PCS; SF-12 Physical Health Composite Score. KT; kidney transplant.
